# Increased Acid-Producing Diet and Past Smoking Intensity Are Associated with Worse Prognoses among Breast Cancer Survivors: A Prospective Cohort Study

**DOI:** 10.3390/jcm9061817

**Published:** 2020-06-11

**Authors:** Tianying Wu, Fang-Chi Hsu, John P. Pierce

**Affiliations:** 1 Division of Epidemiology and Biostatistics, School of Public Health, San Diego State University, San Diego, CA 92182, USA; 2 Moores Cancer Center, School of Medicine, University of California, San Diego, CA 92093, USA; jppierce@ucsd.edu; 3 Department of Biostatistics and Data Science, Division of Public Health Sciences, Wake Forest School of Medicine, Winston-Salem, NC 27157, USA; fhsu@wakehealth.edu

**Keywords:** dietary acid load, smoking status, pack-years, breast cancer survival, mortality, recurrence

## Abstract

Current dietary guidelines do not consider cancer survivors’ and past smokers’ low capacity to regulate their acid–base balance. People with a low capacity to regulate their acid–base balance are more susceptible to acid-producing diets. We studied a cohort of 2950 early stage breast cancer survivors who provided dietary information at baseline and during follow-up. We assessed the intakes of acid-producing diets via two commonly used dietary acid load scores: potential renal acid load (PRAL) and net endogenous acid production (NEAP). We assessed past smoking intensity by pack-years of smoking. After an average of 7.3 years of follow-up, there were 295 total deaths, 249 breast cancer-specific deaths, and 490 cases of recurrent breast cancer. Increased intakes of dietary acid load and pack-years of smoking were each independently and jointly associated with increased total mortality and breast cancer-specific mortality; tests for trends and overall associations were statistically significant for NEAP and marginally significant for PRAL. Compared to women in the lowest tertile of NEAP and pack-year of smoking = 0, women in the highest tertile of NEAP and pack-years of smoking >15 had the greatest increased risk of total mortality (HR = 3.23, 95%CI 1.99–5.26). Further, dietary acid scores were associated with increased breast cancer recurrence among women with pack-years of smoking >0 but not in those with pack-years of smoking = 0 (*p* values for interactions <0.05). Our study provides valuable evidence for adding dietary acid load scores to dietary guidelines for breast cancer survivors and developing specific guidelines for past smokers among these survivors.

## 1. Introduction

Cancer survivors, especially breast cancer survivors, experience accelerated aging due to the detrimental effects of cancer treatments and cancer cells as well as some additional existing risk factors among them [1,2,3]; thus, understanding the risk factors associated with mortality will help design efficient and specialized care for cancer survivors. Breast cancer is the most common type of cancer in women in the United States [4]. Both diet and past smoking history have been found to play important roles in breast cancer prognosis [5,6]; however, current dietary guidelines are not specifically tailored to breast cancer survivors or breast cancer survivors who are past smokers. Further, there is a paucity of research examining the joint impacts of diet and past smoking history on breast cancer.

Dietary acid load may play important roles in breast cancer prognosis and may interact with past smoking intensity in the development of worse prognoses in breast cancer survivors. The proper balance between the acids and bases (i.e., the pH) in the human body is crucial for normal physiologic functions and cellular metabolism [7,8,9]. Acid-producing diets have been found to be associated with cardiovascular-specific mortality [10,11] among healthy cancer-free populations in cohort studies. However, prospective cohort studies examining the associations of acid-producing diets with mortality among cancer survivors are limited. Cancer survivors have a reduced capacity to adjust their acid–base balance [12] and thus they may be more susceptible to acid-producing diets. Furthermore, among cancer survivors, past smokers have a lower capacity to adjust their acid–base balance than never smokers acidosis [13,14,15]. Past smokers accounted for up to 35–40% of breast cancer survivors, whereas current smokers only accounted for 4–6% [16,17,18]. Past smokers with a high intensity of past smoking history have a greater than 50% higher risk of death than never smokers among breast cancer survivors [16,19]. Thus, it is important to compare whether dietary acid load has differential adverse impacts on prognosis in past and never smokers. 

We will leverage a large cohort of breast cancer survivors, the Women’s Healthy Eating and Living (WHEL) study, to conduct the current study. The range of acid-producing diets in this cohort is wider than that of the typical American diet [15], which enabled us to better evaluate the dose–response relationship. Pack-years of smoking, which can accurately assess smoking intensity, was also assessed in this cohort. This study aims to determine whether acid-producing diets and past smoking intensity are risk factors of total mortality, breast cancer-specific mortality, and breast cancer recurrence among early stage breast cancer survivors who were never smokers and past smokers at enrollment. Importantly, we will determine whether past smoking intensity can have a joint impact with an acid-producing diet or modify the impact of an acid-producing diet on breast cancer prognosis. 

## 2. Materials and Methods

### 2.1. Study Design and Population

This study leverages an existing prospective cohort, the WHEL study, comprising mainly early stage (stage I, II, or IIIA) breast cancer survivors. Between 1995 and 2000, the WHEL study enrolled 3088 women within 4 years of diagnosis. The WHEL study was initially a multi-site randomized trial including several sites in the U.S. (i.e., California, Arizona, Texas, and Oregon). The trial was designed to test whether a diet low in fat and rich in vegetables, fruit, and fiber improved breast cancer prognosis. Extensive details regarding inclusion and exclusion criteria can be found in previous publications [20]. Briefly, inclusion criteria were as follows: women who had stage I (≥1 cm), II, or IIIA breast cancer diagnosed within the previous 4 years, had no evidence of cancer recurrence, had completed primary therapy, were 18–70 years old at diagnosis, did not have life-threatening comorbidities, and were able to communicate dietary data via 24-h food recall. Exclusions included insulin dependence and the diagnosis of a comorbidity requiring a specific diet or the use of a medication that contraindicated a high-fiber diet and insulin dependence. Women with diagnoses after age 70 and those with stage 1 tumors smaller than 1 cm were also excluded. 

The initial study used several ways to identify potential participants by using tumor registries as well as communicating and distributing information to community oncologists. Participants completed a run-in period so investigators could identify who might have difficulties adhering to the study requirements. This cohort had five clinical visits in different years, while baseline, year 1, and year 4 had more complete information related to diet. The WHEL study developed an adherence score. The WHEL Feasibility Study indicated that more than 80% of participants reached an adherence score of 450 (full adherence score was 600). This initial trial provided telephone counseling and group cooking classes to the intervention group. Extensive communication programs were implemented to build support and motivate participants to complete the program. For instance, participants could receive an airline ticket as an incentive based on the points earned. Nutritional summaries were also provided to participants after the dietary assessments [20,21].

The intervention did not significantly change breast cancer prognosis after an average of 7.3 years of follow-up. Therefore, the present study considered and analyzed the study sample as a single cohort while controlling the initial trial assignment. For this analysis, we excluded women who were current smokers at baseline; as a result, the analytical cohort comprised 2950 women. 

The Institutional Review Board (IRB) at the University of California at San Diego approved the original study. All subjects provided written informed consent. The de-identified data were provided by the principal investigator of the WHEL study. The current study was an ancillary study using the de-identified data from the WHEL study; thus, the exempt IRB was approved by the San Diego State University IRB committee (protocol number: Temp-1286).

### 2.2. Dietary Assessment

At baseline, year 1, and year 4, dietary intakes were assessed by four prescheduled, 24-h dietary recalls collected by telephone on random days over a 3-week period: two on the weekends and two during weekdays. Dietary assessors used the multi-pass, software-driven recall protocol of the Nutritional Data System software (NDS-R, 1994–2006, 91 University of Minnesota, Minneapolis, MN, USA).

In terms of the assessment of acid-producing diets, two commonly used dietary acid load scores were used to estimate dietary acid load in epidemiological studies: the potential renal acid load (PRAL) score and the net endogenous acid production (NEAP) score. The PRAL score considers the intestinal absorption rates for contributing nutrient ionic balances for protein, potassium, calcium, and magnesium and the dissociation of phosphate at pH 7.4 [22]. Frassetto et al. [23] developed the NEAP score, which uses total protein and potassium intake as the main components involved in acid production. PRAL and NEAP scores were derived from estimations of several nutrient intakes as follows [24]:

PRAL (mEq/day) = (0.49 × protein (g/day)) + (0.037 × phosphorus (mg/day)) − (0.021 × potassium (mg/day)) − (0.026 × magnesium (mg/day)) − (0.013 × calcium (mg/day))

NEAP (mEq/day) = (54.5 × protein (g/day)/potassium (mEq/day)) − 10.2

This study used both scores for dietary acid assessment because they reflect slightly different nutritional intakes and biological mechanisms. A negative PRAL value reflects an alkaline-forming potential; a positive value reflects an acid-forming potential [25]. For NEAP, there is large variation in the general population (ranging from 10 to 150 mEq/day), although a typical Western diet has been characterized by a NEAP score of approximately 50 mEq/day [23,26].

### 2.3. Smoking Assessment

A brief smoking history questionnaire was administered to participants at baseline. The questionnaire included age of smoking initiation and cessation, duration of smoking, and the number of cigarettes/day. We classified a lifetime history of <100 cigarettes as never smoking. Former smokers reported having quit at this baseline survey. All ever smokers reported their intensity of smoking (cigarettes/day) and the number of years they smoked regularly. Pack-years exposure was determined by multiplying duration of smoking by intensity. One pack-year is equal to smoking one pack per day for one year or two packs per day for half a year for 4–6% [16]. 

### 2.4. Assessment of Study Outcome

The primary outcome of this study is total mortality, breast cancer-specific mortality, and breast cancer recurrence. At the close of the study in June 2006, vital status was known for 96% of the participants. Information on death and recurrence from participants was ascertained via confirmation interviews, periodic reviews (including with a family member), and oncologists’ reviews of the medical record and/or death certificate. In addition, both the Social Security and the National Death Index were searched using the Social Security number, name, and date of birth. Causes of death were coded using the International Classification of Diseases, 9th Revision (ICD-9) codes. All breast cancer deaths or recurrences were confirmed by the study’s oncologist. Breast cancer recurrence was defined as the combination outcome of breast cancer recurrence (local, regional, or distal) or new primary breast cancer. Carcinoma in situ was not counted as a study outcome. Survival was assessed as the time from study entry to death or the most recent available review of the Social Security Death Index (updated until 2009). Follow-up time was censored at the earlier of (a) time of the last documented staff contact date or (b) study completion (June 2006) for participants without an event (median follow-up time was 7.3 years, range was 0.01–11.2 years). Approximately four percent of study participants were lost during follow-up and these were censored at the date of last contact.

### 2.5. Other Assessments

Demographic characteristics and health status, including a series of comorbid conditions (e.g., diabetes, cardiovascular diseases, digestive conditions, arthritis, osteoporosis, and medications such as diabetic, cardiovascular, and digestive medications), were self-reported.

Variables abstracted from patient records included initial cancer diagnosis and treatment. Specific variables abstracted included tumor stage, size, hormone receptor status, and use of radiation, chemotherapy, and/or post treatment anti-estrogens use. Physical activity levels were assessed using an adapted validated questionnaire from the Women’s Health Initiative [27]. Physical activity was converted into metabolic equivalent tasks (METs), as previous studies did [28]. 

### 2.6. Statistical Analyses 

Differences in baseline characteristics across breast cancer prognosis or across baseline dietary acid load were evaluated using a *t*-test and analysis of variance (ANOVA) for normally distributed continuous variables, Wilcoxon rank-sum test and Kruskal–Wallis test for non-normally distributed continuous variables, and χ2 test for categorical variables. 

We used Cox proportional hazard models to assess the association of dietary acid load with total mortality, breast cancer-specific mortality, and breast cancer recurrence. Censored times for participants who did not die were calculated from the study entry to lost to follow-up or to the end of the follow-up period, whichever came first. Event time was calculated with respect to each outcome as follows. For all-cause mortality, time was calculated from the study entry to the time of death. For breast cancer-specific mortality, time was calculated from the study entry to breast cancer-specific death; death from other causes was treated as a competing risk. For recurrent breast cancer, time was calculated from the study entry to diagnosis with recurrent breast cancer, and death was treated as a competing risk.

As previously introduced, dietary acid load was characterized by PRAL and NEAP scores. Repeated measures of PRAL and NEAP at years 0, 1, and 4 were analyzed as time-varying covariates. PRAL and NEAP scores were classified into quartiles using the average intakes at years 0, 1, and 4 to set up the cut-point for each quartile. We classified baseline pack-years of smoking into three categories (i.e., 0, 0–15, and 15+). We controlled the following covariates based on a priori assumption: age at diagnosis, race/ethnicity, education level, intervention group, menopausal status, total calorie intake, alcohol intake, smoking status, pack-years, physical activity, body mass index (BMI), education level, tumor stage, tumor size, estrogen and progesterone receptor status, type of anti-estrogen therapy, radiotherapy, chemotherapy, study site, and medical comorbidities. Among these covariates, time-varying covariates included BMI, physical activity, smoking status, total calorie intake, alcohol intake, and types of anti-estrogen therapy. We used baseline data for other covariates. 

We further evaluated both joint impacts of dietary acid load and past smoking intensity on outcomes and effect modification by past smoking intensity. To evaluate joint impacts, dietary acid load was categorized by tertile to improve the stability of point estimates. Women with the lowest tertile of dietary acid load and pack-years of smoking = 0 were treated as the reference group. To evaluate the effect modification by smoking intensity, we conducted stratified analyses by two pack-years of smoking strata (=0 and >0). To assess whether a significant interaction occurred between dietary acid load and pack-years of smoking, we used the Wald *p*-value for the interaction term in a model that also included the main effects. 

The proportional hazards assumption was examined and satisfied in all Cox proportional hazard regression models by testing the significance of the product terms for our variable of interest and log time. All analyses were conducted using SAS version 9.2 (SAS Institute, Cary, NC, USA). 

## 3. Results

### 3.1. Baseline Characteristics by Disease Outcomes in the Whole Cohort 

After a median 7.3 years of follow-up, 295 deaths and 249 breast cancer-specific deaths as well as 490 breast cancer recurrences were reported in the cohort (Table 1). Compared to living group, women who died from all causes tended to have lower proportions of normal weight, above-college education, pack-years of smoking = 0, and positive estrogen receptor (ER) or progesterone receptor (PR) status; they also tended to have higher proportions of women on chemotherapy and higher clinical stage (stage II and stage IIIa). The death group also tended to have lower levels of physical activities. Compared to the living group, women who died of breast cancer tended to have similar patterns to that of the all-cause mortality group except for the tamoxifen users, who tended to have a lower percentage than women who died of breast cancer. Compared to the non-recurrent group, the breast cancer recurrent group tended to have lower proportions of women who had an above-college education, were in menopause, had a positive ER or PR status, and were on tamoxifen; they also tended to have higher proportions of women on chemotherapy and higher clinical stage (stage II and stage IIIa). *p*-values were <0.05 for these comparisons.

### 3.2. Baseline Characteristics by Dietary Acid Load in the Whole Cohort

As shown in Table 2, compared to women with a low dietary acid load, women with a higher dietary acid load were younger and had a lower proportion of White women, postmenopausal women, positive ER or PR status women, and tamoxifen users; they included higher proportions of obese and overweight women and were likely to have less education and engage in less physical activity. *p*-values were <0.01 for these comparisons.

### 3.3. Dietary Acid Load, Past Smoking Intensity, and Risk of Total Mortality for Breast Cancer-Specific Mortality and Breast Cancer Recurrence 

As shown in Table 3, the positive associations of dietary acid load with total mortality and breast cancer-specific mortality were statistically significant for NEAP and marginally significant for PRAL; however, no significant association was found between dietary acid load and breast cancer recurrence. The hazard ratios (HR) comparing the highest to the lowest quartiles of NEAP were 1.54 (95% confidence interval (CI) 1.04–2.29) for total mortality and 1.52 (95%CI 1.01–2.32) for breast cancer-specific mortality; *p*-values for trends were <0.05 for both outcomes. The corresponding HRs for PRAL were similar but marginally significant. Pack-years of smoking was positively and statistically significantly associated with the three outcomes. 

### 3.4. Joint Impact of Dietary Acid Load and Past Smoking Intensity on Breast Cancer Prognosis

We found statistically significant joint associations of dietary acid load and past smoking intensity with total mortality, breast cancer-specific mortality, and breast cancer recurrence (see Table 4). Both dietary acid load scores and smoking intensity appeared to be positively associated with total mortality. Compared to women in the lowest tertile of dietary acid load and pack-year category (pack-year of smoking = 0), women in the highest tertile of dietary acid load and pack-year category (pack-years of smoking >15) had the greatest increased risk of total mortality (HR = 2.86, 95%CI 1.73–4.74 for PRAL; HR = 3.23, 95%CI 1.99–5.26 for NEAP). *p*-values for trends were <0.01 for both PRAL and NEAP. We also observed that the positive associations between dietary acid load and total mortality were stronger in the highest category of pack-years of smoking (>15) than the lower two categories of pack-years of smoking (0 and 0–15). Similar patterns were observed for breast cancer-specific mortality and recurrence; however, the magnitudes and significance were attenuated for recurrence (*p*-values for trends were 0.1 for both PRAL and NEAP).

### 3.5. Stratified Associations of Dietary Acid Load with Disease Outcomes by Past Smoking Intensity

In Table 5, we observed stronger positive associations of dietary acid load with total mortality, breast cancer-specific mortality, and breast cancer recurrence in strata with pack-years of smoking >0 than strata with pack-years of smoking = 0. The positive associations tended to be stronger for NEAP (*p*-values for interactions were 0.1 for total mortality, 0.03 for breast cancer-specific mortality, and 0.01 for breast cancer recurrence).

## 4. Discussion

In these comprehensive analyses of a cohort of breast cancer survivors, increased dietary acid load and past smoking intensity were both independently and jointly associated with increased total mortality and breast cancer-specific mortality. We also found an increased risk of breast cancer recurrence among women with pack-years of smoking >0.

Our study is the first to highlight the importance of the independent and joint impacts of dietary acid load and past smoking intensity on total mortality, breast-cancer specific mortality and recurrence among early stage breast cancer survivors. Previous prospective studies have demonstrated that the dietary acid load or higher metabolic acid load (measured by lower serum bicarbonate and overnight fasting urine pH) had a positive or U-shaped relationship with total mortality or cardiovascular mortality but not with cancer-associated mortality [10,29,30]. These studies followed apparently healthy individuals without cancer at baseline [10,29,30]; thus, whether dietary acid load is associated with total and cancer-specific mortality among cancer survivors cannot be concluded from these studies. Dietary acid load has been shown to increase the risk of hypertension, diabetes, chronic kidney diseases, and hip fractures in cohort studies [31,32,33,34,35]; all of these are risk factors for total mortality [36,37,38]. Furthermore, animal studies have shown that metabolic acidosis can lead to increased cancer development and metastasis [39]. The following discussion helps explain some of the mechanisms. Cancer or cancer treatment itself can damage our bodily systems (electrolytes, respiratory system, kidneys or bone) that need to adjust the acid–base balance [12]. Further, among cancer patients, adaptation to acidosis, in conjunction with oncogenic mutations, endows cancer cells with increased fitness for survival [40]. An acidic microenvironment suppresses antitumor immune responses [41,42] and facilitates treatment resistance [43]. 

We propose the following to help explain why NEAP was found to be a better predictor of breast cancer prognosis than PRAL in our study. Both PRAL and NEAP have their own advantages and disadvantages. NEAP is an approximate of renal net acid excretion (RNAE) and was found to account for 70% of the variation of RNAE [23]. It is a simple estimate of RNAE when compared to PRAL, which considers the roles of other minerals from diets in regulating acid–base balance. PRAL does rely on more information from the dietary database, reduce the predictabilities if the roles of other minerals are negligible, or potentially add measurement errors if the information from other minerals are not accurate or the absorption rates of other minerals varied largely across individuals [26,44]. For instance, Frasseto found that, when adding magnesium to the NEAP score to predict RNAE in 141 healthy subjects (aged 17–73), it actually increased the unaccounted variability by 10% [23]. Moreover, although NEAP and PRAL have strong correlations (r = 0.9), PRAL does not estimate RNAE well when the protein intakes are in low or high ranges [26,44]. It is possible that PRAL has better predictive values than NEAP when minerals other than potassium play important roles in disease development. For instance, PRAL is more predictive of cardiovascular mortality among patients who underwent coronary artery bypass grafting surgery [11] and of depression [45,46]. Other minerals, such as magnesium and calcium, do play important roles in heart functions [47,48] and in the development of depression [49,50,51]. Nevertheless, our study indicates that NEAP, which includes only two key elements (protein and potassium), is an important predictor for total mortality and breast cancer-specific mortality in the whole cohort, and for breast cancer recurrence among past smokers. It also suggests that renal function could be one of the key determining factors in these relationships. Future studies should estimate renal functions to unravel the biological mechanism.

Several mechanisms can explain why past smokers are more susceptible to dietary acid load than never smokers. Quitting smoking can avoid further damage but does not remove past damages by smoking [16,52]; smoking can further damage the acid–base regulating systems [53,54,55,56] and can promote acidosis in patients with or without cancer [13,14]. All of these factors help explain the mechanisms associated with the accelerated risk of total mortality, breast cancer-specific mortality and recurrence for past smokers among breast cancer survivors. 

The original intervention was a low-fat, high-fruit, and -vegetable dietary intervention, not an intervention aiming to change dietary acid load scores, though we did find that dietary acid load scores were significantly lower in the intervention group than the control group. One may question why dietary acid load was found to have a significant impact on prognosis whereas the original intervention did not have a significant impact on prognosis. There are several possible reasons for this. Dietary acid load scores were greatly reduced in the intervention group when compared to the control group; however, 17% and 53% from the intervention group still had high NEAP scores (higher than median) at year 1 and 4, respectively, as compared to 54% and 63% from the control group. Such a mismatch can significantly attenuate the association if it were analyzed as a clinical trial. In addition to dietary acid load, types of fat and intakes of refined carbohydrates were not controlled in the original WHEL intervention. As we know, diets with high intakes of trans fatty acids and glycemic load can increase inflammation and hyperglycemia [57,58], which are risk factors for mortality and cancer recurrence [36,59,60,61,62].

This study has several strengths. It is the first large prospective cohort study investigating the independent and joint associations of dietary acid load and past smoking intensity with breast cancer prognosis among breast cancer survivors. Four 24-h recalls during each visit (baseline, year 1, and year 4) were the unique advantages of this cohort but were rarely conducted in other cohorts. Such advantages enable us to assess dietary acid load more accurately and examine its longitudinal relationships with prognosis outcomes. As this study was originally a trial of high-vegetable, high-fruit, and low-fat intake interventions, we observed a wider range of dietary acid load than other cohorts. This study assessed pack-years of smoking, which can better evaluate past smoking intensity than smoking status. The large sample size provided us with sufficient power to adjust for multiple covariates. However, this study also has limitations. This cohort’s follow-up time was relatively short, and this cohort was comprised predominantly of White women, which will not allow us to examine long-term impacts or generalize our results to other ethnic groups. There was also the possibility of volunteer bias, as the women in the WHEL cohort were highly motivated, health-conscious breast cancer survivors. HER2 status was a not available for the WHEL participants, which may have resulted in residual confounding. WHEL participants were enrolled up to 4 years after their initial diagnosis; thus, the study may not represent breast cancer patients who recur shortly after diagnosis.

## 5. Conclusions

Current dietary guidelines, such as the American Cancer Society’s (ACS) dietary guidelines for breast cancer survivors, do not include dietary acid load and specific dietary guidelines for former smokers [63,64]. Increased dietary acid load and past smoking intensity are both independently and jointly associated with increased total mortality, breast cancer-specific mortality, and recurrence. Monitoring and reducing the protein-to-potassium ratio is important for preventing adverse prognoses among breast cancer survivors. Our results provide important messages for clinicians and dietitians. Precision care and individualized nutrition for breast cancer survivors are important emerging trends. Our results provide valuable evidence for modifying current ACS dietary guidelines regarding dietary acid load and offer specific guidelines for past smokers with different past smoking intensities.

## Figures and Tables

**Table 1:** Baseline characteristics of breast cancer survivors by breast cancer recurrence, total mortality and breast cancer specific mortality (n = 2950).

	Total Mortality			Breast Cancer Specific Morality			Breast Cancer Recurrence		
	No (N = 2655)	Yes (N = 295)	*p*-Value	No (N = 2655)	Yes (N = 249)	*p*-Value	No (N = 2460)	Yes (N = 490)	*p*-Value
PRAL (mEq/day) ^a^	−3.97 (−14.11 to 4.42)	−2.93 (−13.12 to 5.17)	0.3	−3.97 (−14.10 to 4.42)	−2.52 (−12.59 to 5.85)	0.2	−4.10 (−14.15 to 4.42)	−2.84 (−13.14 to 5.17)	0.1
NEAL (mEq/day)	39.78 (32.25 to 48.22)	40.79 (33.12 to 48.89)	0.3	39.80 (32.21 to 48.22)	41.03 (33.50 to 48.68)	0.3	39.65 (32.08 to 48.22)	40.87 (33.36 to 48.68)	0.2
Basic									
Age at diagnosis (years)	50.0 (45.0–57.0)	51.0 (44.0–59.0)	0.3	50.0 (45.0–57.0)	50.0 (43.0–57.0)	0.2	50.0 (45.0–57.0)	49.0 (42.0–56.0)	0.3
White (%)	85.4	82.4	0.2	85.4	83.1	0.3	85.4	85.5	0.7
Body mass index									
Normal weight (%)	44.0	37.3	0.006	44.0	39.0	0.03	43.5	42.7	0.2
Overweight an obese (%)	56.0	63.7		56.0	61.0		56.4	57.3	
Education, at or above college (%)	56.3	46.8	0.002	56.3	46.4	0.0003	56.3	50.4	0.04
Postmenopausal women (%)	79.2	79.7	0.3	79.2	76.7	0.3	80.2	74.5	0.001
Smoking status									
Past smoker (%)	43.2	48.1	0.1	43.2	47.3	0.2	43.4	43.8	0.9
Never smoker (%)	56.8	51.9		56.8	52.7		56.6	56.2	
Pack-year status									
Pack-years = 0 (%)	56.3	50.8	<0.0001	56.3	51.4	<0.0001	55.7	55.7	0.11
Pack-years >0 to 15 (%)	27.8	21.7		27.8	22.5		27.7	24.9	
Pack-years >15 (%)	14.5	23.4		14.5	21.3		15.1	16.5	
Alcohol abstainer (%)	31.2	35.9	0.1	31.2	35.9	0.1	31.3	33.5	0.5
Physical activity (MET/week)	600 (180–1300)	450 (105–930)	0.001	600 (180–1300)	435 (100–975)	0.003	600 (180–1295)	525 (120–1110)	0.09
Intervention group (%)	49.8	50.2	0.9	49.9	48.6	0.7	49.6	50.1	0.9
Chemotherapy (%)	68.8	80.3	0.0002	68.8	86.8	<0.0001	67.9	80.6	<0.0001
Radiation (%)	61.8	61.4	0.8	61.8	61.9	0.8	61.6	62.5	0.8
Hormone receptor status									
ER+/PR+ (%)	62.9	50.9	0.0002	62.9	47.8	<0.0001	62.9	55.3	0.01
ER-/PR- (%)	21.3	29.8		21.3	32.5		19.1	24.5	
Cancer stage at diagnosis (%)									
I	40.4	20.0	<0.0001	40.4	14.5	<0.0001	41.9	20.2	<0.0001
II	55.5	67.1		55.5	71.1		54.1	69.6	
IIIa	4.2	12.9		4.2	14.5		4.0	10.2	
Tamoxifen use (%)	66.8	61.0	0.1	66.8	57.4	0.009	67.6	59.4	0.001

^a^ Continuous variables are presented as median (inter-quartile range). Abbreviations: PRAL: potential renal acid load; NEAP: net endogenous acid production; METS: metabolic equivalent/week; ER: estrogen receptor positive; PR: progesterone receptor positive.

**Table 2:** Baseline characteristics of breast cancer survivors by quartiles of the baseline PRAL score (n = 2950).

	PRAL Score Quartiles (mEq/day)				
	Quartile 1	Quartile 2	Quartile 3	Quartile 4	*p*-Value
	<−13.7 (n = 771)	−13.7 to <−3.7 (n = 769)	−3.7 to <4.7 (n = 771)	≥4.7 (n = 770)	
NEAL (mEq/day) ^a^	27.4 (23.9–30.7)	36.4 (33.7–38.5)	43.7 (41.1–46.3)	55.4 (50.9–61.3)	<0.001
**Basic**					
Age at diagnosis (years)	52.0 (47.0–58.0)	51.0 (46.0–58.0)	50.0 (45.0–57.0)	48.0 (42.0–55.0)	<0.001
White (%)	89.6	88.7	83.8	78.2	<0.001
Body mass index					
Normal weight (%)	56.6	46.7	37.1	32.8	<0.001
Overweight and obese (%)	43.4	53.3	63.9	67.2	
Education, at or above college (%)	64.8	57.4	52.7	46.3	<0.001
Postmenopausal women (%)	84.5	80.1	80.0	73.2	0.001
Smoking status					
Past smoker (%)	44.6	43.0	44.1	43.1	0.9
Never smoker (%)	55.4	56.9	55.9	56.9	
Pack-year status					
Pack-years = 0 (%)	54.8	56.6	55.3	56.3	0.06
Pack-years > 0 to 15 (%)	28.0	24.6	27.9	28.3	
Pack-years > 15 (%)	15.8	17.7	14.3	13.6	
Alcohol abstainer (%)	32.1	30.5	33.7	30.8	0.3
Physical activity (MET/week)	825 (330–1500)	630 (225–1335)	480 (150–1080)	405 (60–1080)	<0.001
Chemotherapy (%)	63.6	61.4	59.5	62.5	0.3
Radiation (%)	63.6	61.0	59.1	62.2	0.6
Hormone receptor status					
ER+/PR+ (%)	63.2	63.1	62.3	58.1	0.003
ER−/PR− (%)	16.2	18.8	21.7	23.6	
Cancer stage at diagnosis (%)					
I	38.8	36.7	38.7	38.9	0.4
II	55.4	59.6	56.7	55.0	
III a	5.7	3.7	4.6	6.2	
Tamoxifen use (%)	72.0	66.9	63.6	62.2	0.001

^a^ Continuous variables are presented as median (inter-quartile range). Abbreviations: PRAL: potential renal acid load; NEAP: net endogenous acid production; METS: metabolic equivalent/week; ER: estrogen receptor positive; PR: progesterone receptor positive.

**Table 3:** Dietary acid load and past smoking intensity in relation to total mortality, breast cancer-specific mortality and breast cancer recurrence.

		Total Mortality		Breast Cancer-Specific Mortality		Breast Cancer Recurrence	
		Event	HR (95%CI)	Event	HR (95%CI)	Event	HR (95% CI)
**Dietary acid load**							
**PRAL(mEq/day)**	Range						
Quartile 1	<−19.50	40	Ref	34	Ref	61	Ref
Quartile 2	−19.50 to <−6.94	77	1.17 (0.81–1.69)	60	1.08 (0.73–1.54)	133	0.98 (0.76–1.27)
Quartile 3	−6.94 to <3.22	89	1.41 (0.97–2.06)	80	1.43 (0.96–2.13)	147	1.07 (0.82–1.39)
Quartile 4	≥3.22	89	1.30 (0.87–1.94)	75	1.27 (0.83–1.94)	149	1.09 (0.83–1.43)
**P for trend**			0.09		**0.09**		0.5
**NEAP(mEq/day)**	Range						
Quartile 1	<28.44	35	Ref	29	Ref	61	Ref
Quartile 2	28.44 to <37.25	82	1.27 (0.88–1.84)	66	1.27 (0.87–1.87)	127	1.06 (0.82–1.37)
Quartile 3	37.25 to <46.90	86	1.50 (1.02–2.21)	77	1.46 (0.96–2.21)	152	1.01 (0.77–1.32)
Quartile 4	≥46.90	92	1.54 (1.04–2.29)	77	1.52 (1.01–2.32)	150	1.15 (0.88–1.50)
**P for trend**			**0.03**		0.04		0.4
**Past smoking intensity**							
**Pack-year category**	Range						
1	0	150	Ref	128	Ref	273	Ref
2	0–15	64	0.96 (0.71–1.28)	56	1.02 (0.75–1.39)	122	0.96 (0.77–1.17)
3	15+	69	1.71 (1.28–2.31)	53	1.68 (1.23–2.30)	81	1.17 (0.91–1.51)
**P for trend**			<0.0001		0.001		0.03

HRs were derived from Cox proportional hazards regression models adjusted for multiple covariates. Covariates in the Cox model included age at diagnosis, race/ethnicity, education level, intervention group, menopausal status at baseline, total calorie intake, alcohol intake, physical activity, body mass index, number of comorbidities, tumor stage, tumor size, estrogen and progesterone receptor status, tamoxifen use, radiotherapy, and chemotherapy. Pack-years of smoking was always adjusted in the multivariable models, but PRAL and NEAP were not adjusted simultaneously. Abbreviations: HR: hazard ratio; PRAL: potential renal acid load; NEAP: net endogenous acid production; WHEL: Women’s Healthy Eating and Living study.

**Table 4:** Joint associations of dietary acid load and past smoking intensity with total mortality, breast cancer-specific mortality, and breast cancer recurrence.

		PRAL (mEq/day)						NEAP (mEq/day)				
		Tertile 1		Tertile 2		Tertile 3		Tertile 1		Tertile 2		Tertile 3
		<−15.04		−15.04 to <−0.71		≥ −0.71		<31.5		31.5 to <43.4		≥43.4
**Total mortality**												
	N	HR (95%CI)	N	HR (95%CI)	N	HR (95%CI)	N	HR (95%CI)	N	HR (95%CI)	N	HR (95%CI)
Pack-years = 0	32	Ref	60	1.48 (0.98–2.24)	58	1.16 (0.76–1.78)	33	Ref	58	1.39 (0.92–2.08)	59	1.18 (0.76–1.81)
0< Pack-years ≤15	10	1.13 (0.69–1.85)	24	1.01 (0.58–1.77)	30	1.25 (0.74–2.10)	10	1.05 (0.63–1.74)	25	1.10 (0.65–1.86)	29	1.22 (0.72–2.06)
Pack-years > 15	16	1.26 (0.71–2.21)	24	2.20 (1.33–3.66)	29	2.86 (1.73–4.74)	15	1.35 (0.78–2.35)	23	1.67 (0.97–2.88)	31	3.23 (1.99–5.26)
P for trend		0.004						0.0001				
**Breast cancer-specific mortality**												
	N	HR (95%CI)	N	HR (95%CI)	N	HR (95%CI)	N	HR (95%CI)	N	HR (95%CI)	N	HR (95%CI)
Pack-years = 0	25	Ref	50	1.39 (0.89–2.20)	53	1.13 (0.71–1.79)	26	Ref	49	1.20 (0.77–1.89)	53	1.08 (0.68–1.72)
0< Pack-years ≤15	9	1.17 (0.70–1.99)	20	0.92 (0.50–1.70)	27	1.36 (0.78–2.37)	8	1.04 (0.62–1.76)	22	0.99 (0.56–1.75)	26	1.26 (0.72–2.21)
Pack-years > 15	13	1.12 (0.61–2.06)	19	2.08 (1.19–3.63)	21	2.65 (1.54–4.57)	11	1.19 (0.66–2.14)	19	1.48 (0.82–2.68)	23	2.82 (1.67–4.76)
P for trend		0.002						0.002				
**Breast cancer recurrence**												
	N	HR (95%CI)	N	HR (95%CI)	N	HR (95%CI)	N	HR (95%CI)	N	HR (95%CI)	N	HR (95%CI)
Pack-years = 0	56	Ref	106	0.90 (0.68–1.23)	111	0.90 (0.67–1.21)	56	Ref	105	0.97 (0.72–1.30)	112	0.94 (0.69–1.27)
0< Pack-years ≤15	19	0.88 (0.62–1.25)	48	0.88 (0.61–1.28)	55	0.90 (0.62–1.29)	20	0.94 (0.66-1.34)	47	0.80 (0.54–1.17)	55	1.01 (0.70–1.46)
Pack-years > 15	18	0.79 (0.50–1.25)	32	0.97 (0.68–1.52)	31	1.69 (1.10–2.64)	15	0.84 (0.53–1.34)	34	1.03 (0.66–1.59)	32	1.64 (1.09–2.46)
P for trend		0.1						0.1				

HRs were derived from Cox proportional hazards regression models adjusted for multiple covariates. Covariates in the Cox model included age at diagnosis, race/ethnicity, education level, intervention group, menopausal status at baseline, total calorie intake, alcohol intake, physical activity, body mass index, number of comorbidities, tumor stage, tumor size, estrogen and progesterone receptor status, tamoxifen use, radiotherapy, and chemotherapy. PRAL and NEAL were not adjusted simultaneously. Abbreviations: HR: hazard ratio; PRAL: potential renal acid load; NEAP: net endogenous acid production; WHEL: Women’s Healthy Eating and Living study.

**Table 5:** Hazard ratios for total mortality, breast cancer-specific mortality and recurrence associated with dietary acid load in different pack-years of smoking strata.

			Total Morality	Breast Cancer-Specific Morality		Breast Cancer Recurrence
**PRAL (mEq/day)**						
**Pack-Years = 0**	Range	Events	HR (95%CI)	HR (95%CI)	Events	HR (95%CI)
Quartile 1	<−19.50	21	Ref	Ref	38	Ref
Quartile 2	−19.50 to <−6.94	41	1.07 (0.64–1.79)	1.04 (0.58–1.85)	75	0.95 (0.67–1.32)
Quartile 3	−6.94 to <3.22	45	1.35 (0.80–2.29)	1.31 (0.71–2.43)	73	0.94 (0.65–1.34)
Quartile 4	≥3.22	43	1.10 (0.63–1.93)	1.13 (0.60–2.10)	87	0.98 (0.68–1.40)
P for trend			0.6	0.6		0.9
**Pack-Years > 0**	Range	Events	HR (95%CI)	HR (95%CI)	Events	HR (95%CI)
Quartile 1	<−19.50	17	Ref	Ref	21	Ref
Quartile 2	−19.50 to <−6.94	33	1.17 (0.69–1.99)	1.03 (0.56–1.90)	53	0.98 (0.64–1.50)
Quartile 3	−6.94 to <3.22	41	1.45 (0.84–2.49)	1.54 (0.86–2.75)	71	1.34 (0.89–2.03)
Quartile 4	≥3.22	42	1.51 (0.84–2.69)	1.54 (0.79–3.01)	58	1.28 (0.83–1.99)
P for trend			0.1	0.6		0.1
P for interaction			0.4	0.09		0.03
**NEAP (mEq/day)**						
**Pack-Years = 0**	Range	Events	HR (95%CI)	HR (95%CI)	Events	HR (95%CI)
Quartile 1	<28.44	17	Ref	Ref	32	Ref
Quartile 2	28.44 to <37.25	48	1.26 (0.76–2.10)	1.26 (0.72–2.23)	78	1.07 (0.76–1.50)
Quartile 3	37.25 to <46.90	40	1.46 (0.86–2.50)	1.39 (0.74–2.61)	79	0.90 (0.62–1.30)
Quartile 4	≥46.90	45	1.30 (0.75–2.27)	1.29 (0.70–2.39)	84	1.05 (0.73–1.50)
P for trend			0.4	0.6		0.9
**Pack-Years > 0**	Range	Events	HR (95%CI)	HR (95%CI)	Events	HR (95%CI)
Quartile 1	<28.44	16	Ref	Ref	25	Ref
Quartile 2	28.44 to <37.25	32	1.25 (0.74–2.15)	1.20 (0.67–2.13)	47	1.11 (0.72–1.70)
Quartile 3	37.25 to <46.90	41	1.44 (0.82–2.53)	1.38 (0.75–2.56)	68	1.17 (0.76–1.80)
Quartile 4	≥46.90	44	1.81 (1.04–3.16)	1.88 (0.75–2.55)	63	1.45 (0.94–2.40)
P for trend			0.03	0.04		0.09
P for interaction			0.1	0.03		0.01

HRs were derived from Cox proportional hazards regression models adjusted for multiple covariates. Covariates in the Cox model included age at diagnosis, race/ethnicity, education level, intervention group, menopausal status at baseline, total calorie intake, alcohol intake, physical activity, body mass index, number of comorbidities, tumor stage, tumor size, estrogen and progesterone receptor status, tamoxifen use, radiotherapy, and chemotherapy. PRAL and NEAL were not adjusted simultaneously. Abbreviations: HR: hazard ratio; PRAL: potential renal acid load; NEAP: net endogenous acid production; WHEL: Women’s Healthy Eating and Living study.
